# Role of *Bacillus subtilis* Spore Core Water Content and pH in the Accumulation and Utilization of Spores’ Large 3-Phosphoglyceric Acid Depot, and the Crucial Role of This Depot in Generating ATP Early during Spore Germination

**DOI:** 10.3390/microorganisms11010195

**Published:** 2023-01-12

**Authors:** George Korza, Michelle Goulet, Angela DeMarco, James Wicander, Peter Setlow

**Affiliations:** Molecular Biology and Biophysics Department, UConn Health, Farmington, CT 06030-3305, USA

**Keywords:** spores, Bacillus, disinfection, metabolism, 3-phosphoglycerate

## Abstract

The development of Bacillus spore cores involves the accumulation of 3-phosphoglycerate (3PGA) during sporulation, following core acidification to ~6.4, and before decreases in core water content occur due to Ca-dipicolinc acid (CaDPA) uptake. This core acidification inhibits phosphoglycerate mutase (PGM) at pH 6.4, allowing 3PGA accumulation, although PGM is active at pH 7.4. Spores’ 3PGA is stable for months at 4 °C and weeks at 37 °C. However, in wild-type spore germination, increases in core pH to 7.5–8 and in core water content upon CaDPA release and cortex peptidoglycan hydrolysis allow for rapid 3PGA catabolism, generating ATP; indeed, the earliest ATP generated following germination is from 3PGA catabolism. The current work found no 3PGA in those *Bacillus subtilis* spores that do not accumulate CaDPA during sporulation and have a core pH of ~7.4. The ATP production in the germination of 3PGA-less spores in a poor medium was minimal, and the germinated spores were >99% dead. However, the 3PGA-replete spores that germinated in the poor medium accumulated >30 times more ATP, and >70% of the germinated spores were found to be alive. These findings indicate why 3PGA accumulation during sporulation (and utilization during germination) in all the *Firmicute* spores studied can be crucial for spore revival due to the generation of essential ATP. The latter finding further suggests that targeting PGM activity during germination could be a novel way to minimize the damaging effects of spores.

## 1. Introduction

The spores of *Firmicute* species are metabolically dormant, resistant to many stresses, and can survive for hundreds of years [1]. Notably, the spores of some species are vectors for food spoilage and human diseases [2]. Germination is how spores break dormancy and lose resistance [3], thereby generating growing cells that can cause the adverse effects noted above. Consequently, there is much interest in how spores trigger germination, as well as the subsequent major events in spore revival that include outgrowth and then vegetative cell growth [2,3].

Spores have many novel features that result in their dormancy and resistance. One is the core’s low water content, which is as low as 25% wt for a spore in water [4]. This low core water content is achieved by two mechanisms, which are likely initiated by the physical action of the spore cortex peptidoglycan layer as it forms in the developing spore exerting inward pressure on the core and inner membrane (IM), presumably because the inner coat layers are sufficiently rigid that this pressure leads to water extrusion from the spore core and even to the intrusion of some of the IM into the spore core [5,6]. Additionally, following the cortex’s effects, the developing spores take up a 1:1 chelate of Ca^2+^ with pyridine-2,6-dicarboxylic acid (CaDPA) to ~25 wt% of the dry core; the CaDPA is made in the mother cell compartment of the sporulating cell, and CaDPA uptake leads to further loss of core water [4,5]. A second remarkable spore feature is that they contain little, if any, ATP, although they have significant levels of AMP [7,8]. However, the synthesis of ATP following the completion of spore germination allows macromolecular synthesis and the uptake of nutrients and ions [2,7,9]. There are two stages in *Bacillus* spore germination [3]. In Stage I, a variety of germinants trigger germination, and the spores release the H^+^, Na^+^, and K^+^ from the core, concomitant with an increase in core pH. This is followed by the release of the cores’ CaDPA, which further increases core pH to ~7.5–8, while the dormant spore core was initially at 6–6.5 [10,11]. CaDPA release also increases spore core water content, appreciably, in *Bacillus subtilis* spores from 35 to ~45 wt% for the wet core [12]. These Stage I events trigger Stage II, which is initiated by the degradation of spores’ peptidoglycan cortex by two redundant cortex-lytic enzymes (CLEs), CwlJ and SleB. Cortex degradation leads to full core swelling, achieving a core water content of ~80 wt% when wet, which is comparable to that in growing cells, and a pH of 7.5–8. It is only after the completion of germination that rapid enzyme activity begins in the core, and at least one soluble protein is relatively immobile in the relatively dehydrated dormant spore core and in Stage I germinated spores [13]. Consequently, the production of ATP only begins after germination is completed [7,14,15].

Once germination is completed, the spores can use several energy reserves to generate ATP, although these reserves do not generate ATP in dormant spores, even if they are incubated for weeks at 37 °C [16]. One such reserve is small, spore-specific proteins, some of which normally bind spore DNA but dissociate when core water content rises to that in a growing cell [17]. At this time, the endoprotease cleavage of the DNA-free proteins, plus peptidase action, generates a large amount of free amino acids, some of which are used for ATP generation [18]. However, fully accessing this energy reserve requires the completion of spore germination as well as time for proteolysis [12]. In addition, much of the free amino acids generated in this proteolysis are released from the spores [18,19], and their use to generate ATP requires the fully germinated spores to take up these amino acids in a process that almost certainly requires ATP. The second spore energy reserve is the 3-phosphoglyceric acid (3PGA) in the spores of all species that have been examined (5–18 mmol/g dry spores) [8]. 3PGA is accumulated late in sporulation in the developing spore within its mother cell soon after the developing spore pH falls to 6–6.5, while the mother cell pH remains constant at 7.8–8 [10,11,20]. After forespore acidification but prior to CaDPA uptake, the *Bacillus* forespore accumulates 3PGA, likely because the phosphoglycerate mutase (PGM) that interconverts 3PGA and 2PGA to allow 2PGA catabolism via the lower part of the glycolytic pathway is extremely pH-sensitive, and PGM’s activity at pH ~6.5 is minimal [21]; this latter property presumably allows 3PGA accumulation in the low pH environment of the developing spore. Indeed, artificially increasing forespore core pH during sporulation or increasing PGM levels ≥ 10-fold results in much less PGA accumulation in the spores [20]. However, increasing the core pH in dormant spores to ~7.8 has no effect on 3PGA levels, presumably because of the low core water content in mature spores, which is ~35 wt% for the wet core compared to the 45 wt% wet core prior to CaDPA uptake, precludes PGM action [9]. Indeed, spores’ 3PGA depot is stable for weeks when dormant spores blocked by germination are held at 37 °C [16].

Clearly, the low pH of a developing spore’s core is important in 3PGA accumulation during sporulation, and the core pH increase in Stage 1 of germination is also likely significant in 3PGA utilization [9,10]. Notably, during sporulation, the decrease in forespore pH and 3PGA accumulation take place prior to CaDPA uptake, and H^+^ release in Stage 1 germination also precedes CaDPA release, although 3PGA metabolism to generate ATP is minimal, if any, until after germination is complete [12]. Despite what we know about 3PGA accumulation and utilization, major questions remain, including what roles do levels of core CaDPA, water, and pH play in 3PGA accumulation and/or stability in dormant spores or in utilization during Stage I of spore germination and is the ATP generated from 3PGA important in the viability of germinated spores? The latter question was studied years ago using *B. subtilis* spores with 10-fold elevated PGM levels, which only accumulated 15% of the 3PGA in wild-type spores [20]. That study found no differences in spore germination or outgrowth between the low 3PGA spores and wild-type spores in either a rich medium or a poor medium supplemented with glucose and casamino acids, such that it is actually a fairly rich medium. Thus, it is possible that 3PGA may be essential for early ATP production when germination is complete and thus is essential for spore outgrowth in a truly poor medium. In order to study this latter possibility explicitly, we have examined 3PGA accumulation and breakdown during germination, as well as ATP production and spore viability during spore germination in a truly poor medium using the spores of four isogenic *B. subtilis* 168 strains: (1) two wild-type (wt) strains, PS832 a prototrophic laboratory strain, and PS533, PS832 carrying plasmid pUB110; (2) mutant strain PS3406 that does not accumulate CaDPA in sporulation because it cannot make DPA; and (3) mutant strain FB113 which accumulates CaDPA in sporulation and excretes it in germination, but cannot complete germination because of the lack of both redundant CLEs; remarkably, the FB113 spores’ 3PGA depot disappears upon germination, but no ATP is made. Notably, the spores of both mutant strains are fully viable, although they need special conditions to obtain spore germination [22]. The new studies with spores of these strains and previous work give new insight into factors that modulate 3PGA accumulation in sporulation, its utilization to make ATP immediately upon the completion of spore germination, and perhaps most importantly, the essentiality of ATP derived from 3PGA in maintaining spore viability during germination in a truly poor medium.

## 2. Materials and Methods

### 2.1. B. subtilis Strains Used, and Spore Preparation and Purification

*B. subtilis* strains used in this work are isogenic derivatives of strain 168: (i) two wt strains, PS832 (a laboratory 168 strain), and PS533; PS832 with plasmid pUB110 [23]; (ii) PS3406 [24] that lacks the *spoVA* operon essential for DPA synthesis in the mother cell compartment of the sporulating cell, and the *sleB* gene encoding a cortex-lytic enzyme (CLE) that degrades spores’ peptidoglycan cortex in CaDPA-less spores, triggering rapid spore germination, but because of the *sleB* mutation, PS3406 makes stable CaDPA-less spores, and (iii) FB113, which lacks spores’ two redundant CLEs, SleB and CwlJ [12]. Spores were prepared on 2xSG medium agar plates without antibiotics, as described [5,25]. After 2–3 d of incubation at 37 °C, spores were scraped into 4 °C water and purified by multiple rounds of sonication and centrifugation over 2–4 d, with a final centrifugation through a solution of ~50% Histodenz, in which the CaDPA-replete wt spores pellet and the debris floats [26]. PS3406 spores have a lower core wet density than CaDPA-replete spores, so 45% Histodenz was used. Spores used in this work were >98% free of growing or sporulating cells, germinated spores, or visible debris and were stored at 4 °C, protected from light at an Optical Density at 600 nm (OD_600_) of ~10.

### 2.2. Extraction and Quantitation of Low mol wt Compounds in Spores

The dormant spores of *B. subtilis* strains, 75–125 mg dry wt, were extracted with boiling 80% 1-propanol, the suspension was flash evaporated, small molecules were dissolved in water, the suspension was centrifuged, the supernatant fluid was processed to remove the Mn^2+^ ions, and the samples were prepared for a run on ^31^P-NMR or ^13^C-NMR, as described [16,27]. In one experiment, ~125 mg of FB113 spores were germinated for 2 h at 37 °C in 12.5 mL of 25 mM K-Hepes buffer (pH 7.4) and 10 mM L-valine; prior to germination, the spores were heat-shocked in water for 30 min at 70 °C and then cooled. The germinated spores were harvested by centrifugation, the pelleted spores in 100 μL 20% Histodenz were layered on 2 mL of 45% Histodenz and centrifuged to remove any ungerminated spores, which pellet, while the germinated spores float. Floating material was collected and centrifuged in water several times to remove the Histodenz, and the final purified germinated spores were extracted, processed, and analyzed by ^31^P- and ^13^C-NMR, as described above.

### 2.3. Spore Core pH Determination

The determination of the core pH was measured via ^14^C-methylamine incorporation into the intact spores, as described [9]. Spores of various strains (~15 mg dry wt/mL) in 200 mM Tris-HCl buffer (pH 8.8) were incubated at 24 °C with 5 mM ^14^C-methylamine (Moravek Biochemicals, Brea, CA USA) (~2.5 × 10^5^ dpm ml^−1^). At various times, 600 μL samples were rapidly passed through a 0.22-micron syringe filter; the filtrate was saved, and the filter was washed with two 0.5 mL aliquots of 4 °C 200 mM Tris-HCL buffer (pH 8.8), and then the filtrates saved. All filtrates were made 50 g/L in trichloroacetic acid (TCA), and 600 μL of the TCA was added above the filter. After incubation overnight to elute ^14^C-methylamine from the spores, the filter was centrifuged to collect the eluate, and 500 μL of the eluate, as well as 500 μL of the initial filtrate and wash, were individually added to 4 mL of scintillation fluid, and the samples were counted in a scintillation counter. The pH values of the spore core were calculated from the maximum accumulation of ^14^C-methylamine relative to that of *B. subtilis* PS533 spores, as the core pH of the *B. subtilis* spores was previously determined by this method as ~6.4 [9]; this value was consistent with the values determined later by using pH-sensitive dye [10], with an even lower value reported in another study using a pH-sensitive fluorescent protein [11]. Spore counting in a Petroff–Hauser chamber was used to correct the relative levels of ^14^C-methylamine uptake for the variations in spore numbers between the samples.

### 2.4. Analysis of ATP and Spore Viability during CaDPA Germination

For the analysis of ATP accumulation following spore germination, the spores of the strains at an OD_600_ of 5 were germinated in 50 mM CaDPA (made up to pH 7.5 with Tris base), and 100 μM L-alanine was used on the FB113 spores to trigger their germination. At various times, 500 μL aliquots were added to 2 mL of boiling 1-propanol; the samples were processed, and ATP was measured via light production by luciferase in a luminometer [7,12] using a commercial kit for the ATP measurements. Aliquots were also examined by phase contrast microscopy to assess the completion of germination or CaDPA release, counting ~100 spores. The analysis of the viability of ~100 spores that had germinated with CaDPA was carried out by staining with BacLight reagent and an examination via fluorescence microscopy to determine the percentage of live (green) or dead (red) germinated spores, while the dormant spores stained poorly, if at all [28].

## 3. Results

### 3.1. Levels of 3PGA and Other Phosphorylated Small Molecules in Various Spores

**Figure 1:**
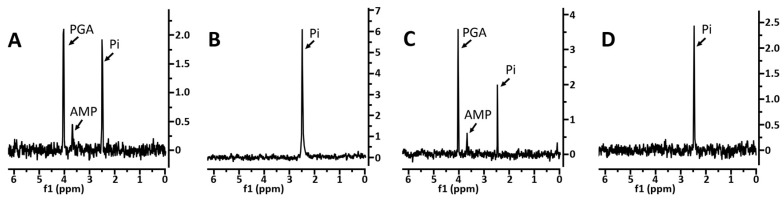
(**A**–**D**) Levels of 3PGA, inorganic phosphate (Pi), and AMP in dormant and germinated spores of various strains as determined by ^31^P-NMR. The spores of various strains, either dormant or germinated, were prepared, extracted, processed, and analyzed by ^31^P-NMR, as described in the Methods section. Extracts analyzed: (**A**) PS533 dormant (1.1 × 10^10^ spores); (**B**) PS3406 dormant (1.9 × 10^10^ spores); (**C**) FB113 dormant (1.5 × 10^10^ spores), and (**D**) FB113 germinated (4 × 10^9^ spores); the identities of all major peaks are labeled; this experiment was carried out twice with nearly identical results.

In the initial experiments, the 3PGA levels were determined in the dormant spores of the strains PS533, PS3406, and FB113 via ^31^P-NMR (Figure 1A–C).

This analysis showed that dormant PS533 and FB113 spores had similar levels of three abundant low mol wt phosphorylated compounds, PGA, AMP, and inorganic phosphate (Pi) (note that different amounts of spores were analyzed), as found previously for the PS533 spores [16], but the CaDPA-less PS3406 spores had no 3PGA, with 2–3 times as much Pi as the other strains and no detectable AMP. These results suggested that 3PGA had either never been accumulated in the developing PS3406 spores or was slowly metabolized in these spores due to their higher core water content. In order to obtain further information about spores’ 3PGA metabolism, the FB113 spores were germinated; the spores that had released CaDPA and, thus, had a core water content ~35% higher than the dormant spores and an elevated core pH were isolated, and the levels of phosphorylated small molecules were measured (Figure 1D). As with the PS3406 spores, 3PGA and AMP were absent from the germinated FB113 spores, and the Pi levels were elevated compared to the total low mol wt phosphate-containing compounds in the dormant FB113 spores (note that lower amounts of the germinated, rather than the dormant spores, were analyzed). The dormant PS3406 and germinated FB113 spore extracts were also analyzed by ^13^C-NMR to look for glyceric acid that was perhaps generated by phosphatase action on 3PGA. However, the levels of glyceric acid were ≤7% of the 3PGA in the dormant PS533 or FB113 spores (data not shown).

### 3.2. Effects of Core pH

**Figure 2:**
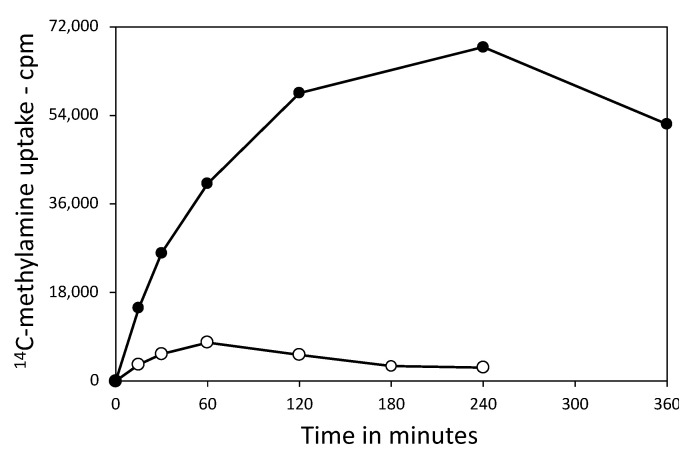
^14^C-Methylamine uptake by the intact spores of strains PS533 and PS3406. Methylamine uptake was measured, as described in the Methods section, and the values have been corrected to compare the uptake via an identical number of spores. The symbols used are PS533—● and PS3406—○.

The absence of 3PGA in the germinated FB113 spores was not surprising as these spores go through Stage I of germination when the core water content rises ~35% and the core pH rises to ~7.5 [9]. However, the lack of 3PGA in PS3406 spores was unexpected, even though they had no CaDPA and an elevated core water content. In order to determine if the PS3406 spores also had a high core pH, we measured the core pH of the dormant wild-type and PS3406 spores by measuring ^14^C-methylamine incorporation into the spore core [9] (Figure 2; Table 1).

The maximum amount of methylamine incorporated into the core of the PS533 spores was consistent with a core pH of ~6.4 (Table 1). However, the maximum amount of methylamine incorporated into the CaDPA-less PS3406 spore core was ~10-fold lower than in the PS533 spores (Table 1). This predicts a core pH in these spores of ~7.4, a pH at which *Bacillus* PGM has high activity [21]. While we do not know when the core pH of the PS3406 spores became ~7.4, given that, after 5–8 h, 3PGA should accumulate in the developing PS3406 spores prior to spore maturation and the release from the sporangium, and given the 2 d needed for spore purification at 4 °C, whenever the core stabilized at a high pH in this period, there would be plenty of time for PGM to facilitate 3PGA utilization. However, since there was also no AMP detected in the germinated FB113 spores, nor any ATP [12] (and see below), and no AMP in the dormant PS3406 spores (again, no ATP, see below), an alternative possibility is that, in the higher core water content of the CaDPA-less spores, even if the core pH was 6.4, some other reaction(s) might have been fast enough to degrade not only the 3PGA but also the AMP releasing Pi. Indeed, the Pi levels in the dormant PS3406 and the germinated FB113 spores are those expected if all Pi was released from 3PGA and AMP, with the values adjusted for the numbers of spores analyzed. These observations further suggest that at least some reactions, presumably those that are enzyme-catalyzed, can take place, albeit perhaps slowly, in the Stage I germinated spores (although not ATP generation).

### 3.3. Effect of 3PGA on ATP Accumulation and the Viability of Germinated Spores

Previous work [7] showed that, after the completion of spore germination, most early ATP generation is from spore energy reserves, specifically from 3PGA. After ATP generation via 3PGA conversion to pyruvate and ATP, and then pyruvate conversion to acetate, NADH, and more ATP [7,30], the other large energy reserve (amino acids) is generated by the proteolysis of a family of spore-specific proteins, but only after 3PGA catabolism begins [18]. Importantly, most of these amino acids are released from spores and must be taken up in ATP-requiring processes.

**Figure 3:**
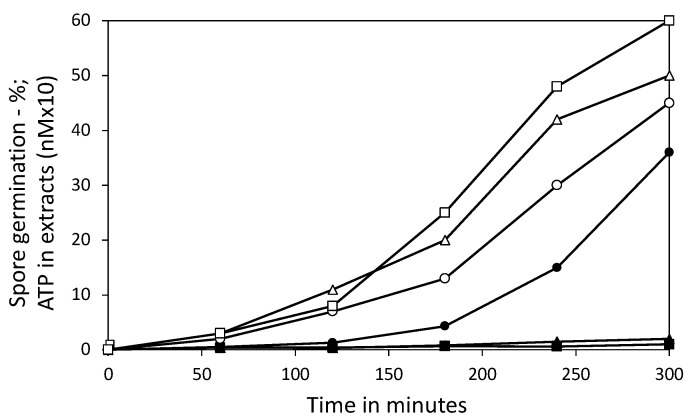
Germination and ATP generation of spores incubated with CaDPA. Strains used: PS832, wt and have 3PGA (○, ●); PS3406, CaDPA-less and no 3PGA (△, ▲); and FB113, can initiate but not complete germination, and have 3PGA (□, ■). Spores were incubated at 23 °C with 50 mM CaDPA (pH 7.5), plus 100 μM L-alanine with the FB113 spores; spore germination (○, △, □) and ATP levels (●, ▲, ■) were measured as described in the Methods section.

**Figure 4:**
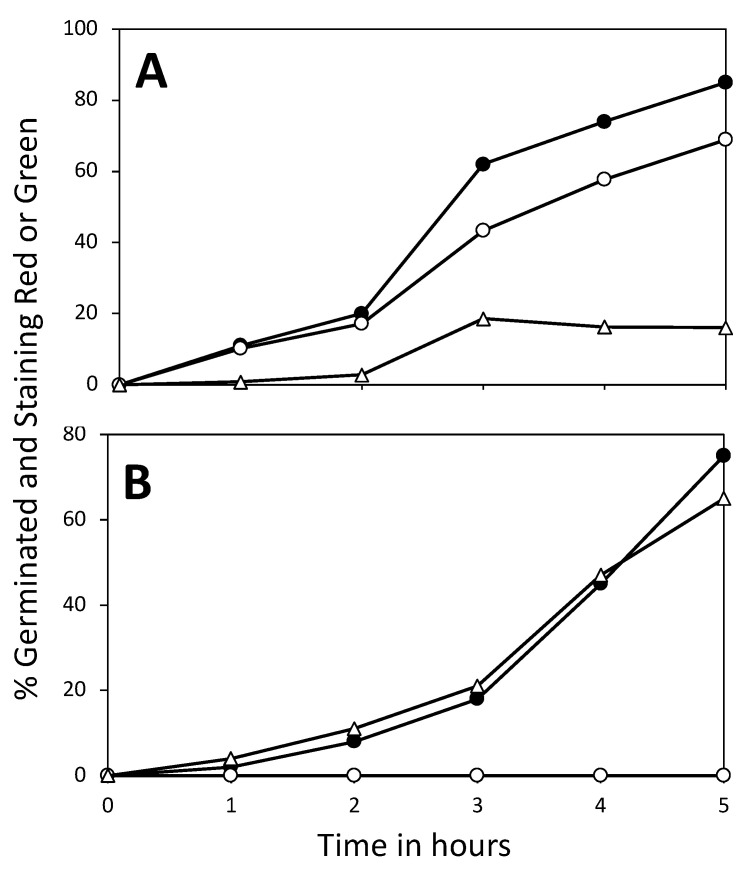
(**A**,**B**) Germination and viability of spores incubated with CaDPA. The spores of the strains (**A**) PS832 (wt and have 3PGA) and (**B**) PS3406 (CaDPA-less and have no 3PGA) were incubated at 23 °C with 50 mM CaDPA (pH 7.5); spore germination was measured by phase contrast microscopy (●) and the levels of germinated spores stained red (△, dead) or green (○, alive) were measured by fluorescence microscopy upon BacLight staining, as described in the Methods section.

In order to examine the importance of 3PGA in ATP generation early in spore germination, we measured ATP generation following the triggering of germination of the PS832, PS3406, and FB113 spores (Figure 3). CaDPA was used as the germinant, as the PS3406 spores do not germinate with physiological germinants [22]. The FB113 spores cannot germinate with CaDPA, as they lack the CLE CwlJ activated by CaDPA [3], so L-alanine was added to 100 μM, with CaDPA present to mimic the environment that the PS832 and PS3406 spores were exposed to. These incubations were examined by phase contrast microscopy to assess the kinetics of the completion of germination of the PS832 and PS3406 spores, which become phase dark. However, the FB113 spores cannot complete germination, so their germination was assessed by phase contrast microscopy to identify those spores that have released CaDPA by observing their lower refractive index. The results of this experiment (Figure 3) indicate that the spores of all three strains germinated, but the PS832 spores accumulated the most ATP, with minimal ATP accumulated by the germinating PS3406 or FB113 spores. The minimal ATP accumulated by the germinated FB113 spores is consistent with previous work [12] that found no generation of ATP in the FB113 spores germinated with high levels of free amino acids. Since 3PGA and AMP disappeared from the germinated FB113 spores (Figure 1D), there must be reactions other than ATP generation in which 3PGA participates in germinating the spores, especially in those spores that only go through Stage I. However, these other reactions are unknown, and there is no evidence of the release of phosphorylated compounds by the germinated spores. The viability of the PS832 and PS3406 spores (germinating in CaDPA) was also measured by BacLight reagent staining, which stains live germinated spores green and dead germinated spores red [28] (Figure 4A,B). As expected from the ATP accumulation results, the CaDPA-germinated PS832 spores were largely stained green (Figure 4A) and were, thus, alive, but >99% of the germinated PS3406 spores were stained red (Figure 4B) and were, thus, dead. An earlier study [20] did find that spores lacking 85% of the wt spore 3PGA level had germinated, gone through outgrowth, and were viable, but the germination medium used was not truly poor, as it contained glucose and amino acids.

## 4. Discussion

The results in this communication further indicate the importance of core water content and pH in the accumulation of major spore reserves of quick energy (3PGA). In addition, if 3PGA is not accumulated in spores or is destroyed prematurely, ATP generation during germination in a nutrient-poor environment decreases greatly, placing 3PGA-less spores at a disadvantage in entering outgrowth because of their lower ability to carry out ATP-requiring processes, such as the uptake of the ions and amino acids released during germination [9,18]. Indeed, the PS3406 spores lacking 3PGA were dead almost immediately after CaDPA germination was complete, while the germinated wild-type spores that had 3PGA were largely alive. These findings about the viability of those germinated spores that did and did not have 3PGA emphasize the importance of a low core pH and water content in developing spores, as this leads to 3PGA accumulation and its stability in spores. This 3PGA depot in spores is an ancient adaptation by *Firmicute* spore-formers, as *Clostridii* spores also accumulate 3PGA, which disappears early during outgrowth [8,27], and, presumably, 3PGA is, again, crucial in providing ATP for these germinated spores as they “return to life”.

The importance of ATP generation from 3PGA soon after spores complete their germination, especially within a poor medium, suggests that this period in spore revival may thus be a period of great vulnerability for germinated spores, as, if sufficient ATP is not generated in this brief period, then the survival of the germinated spores is threatened. Given this information, perhaps blocking 3PGA utilization in germinated spores might be a method for minimizing the levels of the growing cells generated from the spores. In turn, this could reduce the deleterious effects of spores, such as food spoilage, food-borne disease, and serious human diseases or intoxications [2]. Indeed, blocking the lower part of the glycolytic pathway in the germinating spores of *Bacillus megaterium* eliminates ATP production from 3PGA, as well as 3PGA utilization, as *Bacillus* enolase is very sensitive to inhibition by fluoride [7]. However, adding fluoride at levels sufficient to inhibit spore enolase is not something that would be feasible as fluoride also inhibits human enolase. However, another and potentially more specific method to block the production of ATP from 3PGA in germinating spores is to specifically inhibit spore PGM. This approach is possible because there are some major differences between the PGM in *Bacillus* species and the PGM in mammals, as outlined below.

Where studied, the *Bacillus* species have only one PGM and one coding gene; the cell and spore enzymes are identical, and both react identically to an antiserum against the vegetative cell enzyme [31,32]. In addition, the purified *Bacillus* enzyme has a number of specific features, including (i) a strict and specific requirement for Mn^2+^ ions for enzyme activity; (ii) extreme pH sensitivity to enzyme activity (noted in the Introduction section), which is due to the very pH-sensitive binding of Mn^2+^ ions to histidine residues in the enzyme’s active site, and (iii) the lack of any requirement for 2,3-diphosphoglycerate (2,3DPG) for enzyme activity [21,31,32,33,34,35,36]. Notably, the PGMs from at least several *Firmicutes* that do not form spores also require Mn^2+^ are also very pH-sensitive, and do not require 2,3DPG for their activity [34]. In contrast to the specific characteristics of Bacillales species PGM, mammalian PGMs invariably have an absolute requirement for 2,3DPG as a cofactor for catalytic activity; they do not require Mn^2+^ specifically, are not extremely pH-sensitive, and have enzymes that make the 2,3DPG cofactor [37,38]. Notably, while some species, such as plants, generally have only 2,3DPG-independent PGMs, some organisms have both 2,3DPG-independent and -dependent PGMs, including the bacterium *Escherichia coli* [39,40]. Indeed, *B. subtilis* has a gene, *yhfR*, that has significant sequence similarity to 2,3DPG-dependent PGMs. However, partially purified YhfR had little, if any, PGM activity and gave no detectable PGM activity in the extracts of a *B. subtilis pgm* mutant, with or without 2,3DPG [41].

The facts given above indicate that *Bacillus* spores’ PGM is not only quite different from human PGM, but these two enzymes also have different mechanisms of action, despite carrying out the same reaction. Thus, it should be possible to develop an inhibitor of *Bacillus* PGM that would have no effect on human PGM. If so, then such an inhibitor might be able to reduce the fitness of those spores germinating in various conditions such that many die before ever becoming growing cells, thus reducing the spore’s deleterious effects. The fact that the PGM in germinating spores might be a possible drug target is also consistent with recent work, suggesting that one PGM isomer in humans may be a possible drug target in cancer cells [42,43]. Thus, comparable work targeting the Bacillus PGM may lead to a new weapon in the arsenal used against spores.

One finding in this work is left unexplained: what happened to the 3PGA and AMP which were absent in the dormant PS3406 or germinated FB113 spores? One possibility is that, in the developing CaDPA-less PS3406 forespores, 3PGA is used to make some ATP, thus relieving the mother cell from having to provide all the ATP required by the forespore [44], although it is not clear why AMP would be absent as well. Phosphatase action might be another reason for AMP and 3PGA loss from the dormant PS3406 and germinated FB113 spores, which is consistent with the levels of Pi in the PS3406 and germinated FB113 spores. Notably, no glyceric acid was detected in these spores, making the action of a phosphatase alone unlikely unless the spores can slowly metabolize and/or excrete glyceric acid, but currently, there is no complete understanding of the fate of 3PGA in CaDPA-less spores, either during sporulation (PS3406) or germination (FB113). However, the disappearance of 3PGA and AMP from those FB113 spores that complete Stage I of germination but go no further indicates that some reactions can go on in the Stage I germinated spores, although the nature of these reactions is unknown.

## 5. Conclusions

The results of these studies on the spores of these strains, as well as the results from previous work, give new insight into the factors which modulate 3PGA accumulation and utilization to make ATP during spore germination. The importance of 3PGA in preserving spore viability during germination in a poor medium is striking and perhaps indicates why 3PGA accumulation is such an ancient adaptation by spore formers. Finally, the apparent reliance of newly germinated spores on 3PGA to make ATP in poor media, presumably to jump-start their metabolism, may highlight a period of vulnerability for germinating spores when slow 3PGA utilization to make ATP may lead to spore death; if spore PGM could be specifically inhibited, this might lead to a new weapon in the fight against bacterial spores.

## Figures and Tables

**Table 1:** Core pH in spores of several strains *.

Core pH	Spores
6.4	PS533
7.4	PS3406

* Spore core pH was measured based on the maximum ^14^C-methylamine accumulation observed in the experiment in Figure 2 and was calculated as described previously [9,29]. The value calculated for the PS533 spores is essentially identical to that determined for the wild-type *B. subtilis* spores using a pH-sensitive fluorescent dye [10].

## Data Availability

All data are in the manuscript or can be obtained from the corresponding author upon request.
